# Biography of the Late Dr. Drake

**Published:** 1853-01

**Authors:** 


*Biography of the late Dr. Drake.* — Prof. Gross, of the University of Louisville, has consented to a request made by his colleagues, to prepare a biography of the late Dr. Drake. It will probably be publicly pronounced on some occasion during the winter, and will undoubtedly be published. The duty of preparing the biography of his deceased friend appropriately devolves on Prof. G. They were associated at the commencement of the career of the latter as a teacher, in the Medical College of Ohio, and as colleagues for many years in the University of Louisville, the most intimate relations subsisted between them. Prof. G. will do justice to the memory of the distinguished Drake.

---

We have lately purchased a complete set of "H. S. Day's Fracture Splints," made at Bennington, Vermont. We have also applied some of the splints to use: and we deem it only just to say that they are in most respects equal to any others in use, and in some respects superior. They were evidently made by a practical surgeon, possessing two requisites in good splints which are not always found, namely, *fitness* and *firmness.* They are admirably suited to the purposes for which they are severally designed, and they are substantial. The only objection which we can make to them is, that there are in the "complete set," a redundancy of double inclined planes, and not a fair compliment of straight, extending splints for the thigh. But to some surgeons, who are more in the practice of using double planes than ourselves, this might not be regarded as an objection.

They are for sale by A. I. Mathews, Druggist, at Buffalo. F. H. H.

